# Preeclampsia 2012

**DOI:** 10.1155/2012/586578

**Published:** 2012-07-11

**Authors:** Elosha Eiland, Chike Nzerue, Marquetta Faulkner

**Affiliations:** Renal Division and Department of Obstetrics and Gynecology, Department of Internal Medicine, Meharry Medical College, Nashville, TN 37208, USA

## Abstract

Preeclampsia is a common complication of pregnancy associated with high maternal morbidity and mortality and intrauterine fetal growth restriction. There is extensive evidence that the reduction of uteroplacental blood flow in this syndrome results from the toxic combination of hypoxia, imbalance of angiogenic and antiangiogenic factors, inflammation, and deranged immunity. Women treated for preeclampsia also have an increased risk for cardiovascular and renal disease. At present it is unclear if the increased cardiovascular and renal disease risks are due to residual and or progressive effects of endothelial damage from the preeclampsia or from shared risk factors between preeclampsia and cardiac disease. Moreover, it appears that endothelin-1 signaling may play a central role in the hypertension associated with preeclampsia. In this paper, we discuss emerging data on the pathogenesis of preeclampsia and review therapeutic options.

## 1. Introduction

Preeclampsia, a human-pregnancy-specific disease defined as the occurrence of hypertension and significant proteinuria in a previously healthy woman on or after the 20th week of gestation, occurs in about 2–8% of pregnancies [1, 2]. It is the most common medical complication of pregnancy whose incidence has continued to increase worldwide, and It is associated with significant maternal morbidity and mortality, accounting for about 50,000 deaths worldwide annually [3, 4]. Thus reducing maternal mortality by 75% between 1990 and 2015 has been considered as part of the millennium development goals of the World Health Organization (WHO) Nations [5]. Risk factors for preeclampsia include nulliparity, multifetal gestations, previous history of preeclampsia, obesity, diabetes mellitus, vascular and connective tissue disorders like systemic lupus erythematosus and antiphospholipid antibodies, age >35 years at first pregnancy, smoking, and African American race. Among primiparous women, there is a disparity among ethnic groups as the risk in African American women is twice that of Caucasian women, and the risk is also very high in women of Indian and Pakistani origin [6]. The connection between these risk factors and preeclampsia is poorly understood. The differences in risk among ethnic groups suggest a strong role for genetic factors in the pathogenesis of preeclampsia. Most theories on the etiology of preeclampsia suggest that the disease is a cascade triggered by combination of abnormal maternal inflammatory response, endothelial cell activation/damage with deranged hemodynamic milieu, and deranged immunity [7–12]. The precise trigger that unifies the deranged vascular, immune and inflammatory responses remains to be elucidated. In this paper, we discuss emerging concepts in pathogenesis of preeclampsia and review therapeutic options.

## 2. Pathogenesis of Preeclampsia and**** Hemodynamic Changes

Preeclampsia is characterized by placental hypoxia and/or ischemia, excessive oxidative stress, in association with endothelial dysfunction. Release of soluble factors from the ischemic placenta into maternal plasma plays a central role in the ensuing endothelial dysfunction that is the most prominent feature of this disease. Recent data have suggested that endothelial dysfunction in preeclampsia results from an antiangiogenic state mediated by high circulating levels of soluble Fms-like tyrosine kinase 1 (sFlt1) and soluble endoglin in concert with low levels of proangiogenic factors like placental growth factor (PlGF), vascular endothelial growth factor (VEGF). The placenta makes sFlt1 in large amounts, but circulating mononuclear cells have also been shown be an extra source of sFlt1 in preeclampsia [9, 10]. High-circulating levels of sFlt1 have been documented in women with preeclampsia [13], and this high level may predate the onset of preeclampsia [12], and the severity of preeclampsia may correlate with the levels of sFlt1 [13]. Thus sFlt1 acts as a potent and inhibitor of VEGF and PIGF by binding these molecules in the circulation and other target tissues, such as, the kidneys. Consistent with these observations, administration of sFlt1 to pregnant rats produces a syndrome that mimics preeclampsia with hypertension, proteinuria, and edema [14], while anti-VEGF therapy in cancer patients has been shown to cause hypertension and proteinuria in cancer patients [15]. Thus the preponderance of evidence shows that excessive sFlt-1 plays a central role in induction of the preeclampsia phenotype as sFLt-1 decreases VEGF binding to its receptor which reduces phosphorylation of endothelial nitric oxide synthase (eNOS) by VEGF an effect which culminates in reduced eNOS [16].

While it has been traditionally assumed that preeclampsia is a self-limited disease that resolves once the baby and placenta are delivered, some studies have shown that this maternal endothelial dysfunction can last for years after the episode of preeclampsia [17, 18]. A recent analysis actually confirmed that a history of preeclampsia is associated with a doubling of the risk for cardiac, cerebrovascular, and peripheral vascular disease compared to women without such a risk factor [19–22]. Furthermore, such women have also been shown to have increased risk for renal diseases, such as, Focal segmental glomerulosclerosis (FSGS) and microalbuminuria. The children born after a pregnancy complicated by preeclampsia have also been shown to be at high risk for complications like diabetes mellitus, cardiovascular disease, and hypertension [23, 24]. The pathogenesis of this increased risk has not been defined, but suggested contributing factors include fetal malnutrition, epigenetic modification, and postnatal growth acceleration [25]. Furthermore, the endothelial dysfunction and other vascular perturbations observed in preeclampsia actually begin early in pregnancy, though the severe vascular outcomes become evident after 20 weeks of gestation which is an important issue that should be considered in designs of therapeutic and prevention interventions. This may even pose a challenge in the entire concept of diagnosing and treating this disorder as an issue that begins after the 20th week of pregnancy.

## 3. Evidence for Inflammation in Pathogenesis of Preeclampsia

Normal pregnancy induces changes in maternal physiology to accommodate the fetus and the placenta, as well as products from the fetoplacental unit, such as, placental exosomes, microparticles, and microchimeric cells. In normal pregnancy, there is a shift towards a Th2-type immune response which protects the baby from a Th1-type (cytotoxic) response which could harm the baby with its products like interleukin-2, IL-12, interferon *γ* (*IFNY*), and tumor necrosis factor *α* (TNF*α*). Thus inflammation appears to be the link between the adaptive immune response and the occurrence of preeclampsia. Systemic inflammation in preeclampsia appears to favor a preponderance of Th1-type reaction [26]. Redman et al. initially proposed that preeclampsia arises from an exaggerated maternal vascular inflammatory response [27]. Consistent with this observation, some studies show an abundance of soluble markers of neutrophil activation in preeclampsia [28–30], while others have demonstrated amplification of inflammation in preeclampsia by activation of the complement system [31]. Specifically, the cytokines TNF and interleukin 6 (IL-6) are elevated in preeclamptic women. However, the role of inflammation as the cause of preeclampsia is weakened by the failure of several studies to find a strong and consistent association between increase in inflammatory status and clinical signs of preeclampsia [32, 33]. Consistent with these observations, pregnant women with excessive inflammation and high cytokine levels, like those with severe infections, do not always develop preeclampsia.

## 4. A Unifying Hypothesis on Preeclampsia: ****Deranged Endothelin and Nitric ****Oxide Signaling

The placenta is the most central organ in the pathogenesis of preeclampsia. Except for postpartum cases, removal of the placenta abolishes the disease. Furthermore, the placenta, not the fetus is a sine qua non for the development of preeclampsia which can also occur in molar pregnancies. A major advance in the understanding of the pathogenesis of preeclampsia is the development of excellent animal models of this disease. Using these models, such as, the uterine perfusion reduction model [34], it has been shown that the occurrence of hypertension, proteinuria, and endothelial dysfunction was associated with raised sFLt1 and elevated preproendothelin levels. Furthermore, administration of endothelin type A receptor antagonist completely normalized the hypertension in this model, whereas this antagonist had no effect in normal pregnant controls [34]. In a second model where animals were administered excess amounts of sFLt1 to mimic preeclampsia, endothelin signaling was increased and coadministration of an endothelin antagonist abrogated this hypertensive response. This suggests that the hypertension associated with excess sFLt1 in preeclampsia is dependent on endothelin signaling [34]. Soluble endoglin (sEng) is another antiangiogenic factor isolated from the placenta and blood of women with preeclampsia. sEng inhibits the binding of transforming growth factor (TGF)-*β* to its receptor and causes down stream down regulation of nitric oxide synthase [35]. Thus the combination of raised sFlt1 and sEng appears to impair nitric oxide generation and activate endothelin-1 signaling with hypertension and aggravation of maternal endothelial dysfunction [36, 37]. Using knockout mice models, it has been shown that mice lacking endothelial nitric oxide (eNOS) have hypertension, insulin resistance, hyperlipidemia, and decreased nitric oxide (NO) production [37–40]. Some, but not all studies, show that NOS3 polymorphisms leading to lower NO production are associated with hypertension [40] and/or preeclampsia. Finally, Li et al. have recently shown that lack of eNOS aggravates the preeclampsia phenotype induced by increased sFLt1 in nonpregnant female mice [41]. While these animal studies cannot be directly extrapolated to humans they illuminate possible mechanisms where endothelin transmission and changes in eNOS that affect endothelial function can contribute to the pathogenesis of this syndrome. Another humoral mediator whose role has been evaluated in preeclampsia is relaxine. Relaxine is a 6-kilodalton peptide hormone secreted by the corpus luteum and circulates in maternal blood during pregnancy. Its administration presence and/or administration during pregnancy leads to rapid and sustained vasodilation in the maternal circulation. Thus it would appear to be an important hormone to assess in a disease associated with vasoconstriction, such as, preeclampsia. The sustained maternal vasodilatory actions seen with relaxine appear to be critically dependent onvascular endothelial growth factors like gelatinases which in turn activates the endothelium (ET)B/nitric oxide vasodilatory pathway [42–44]. However, though relaxin is indeed significantly elevated in the serum of women in late pregnancy, serum relaxin levels have not been shown to influence blood pressure, renal vascular resistance, renal blood flow, or glomerular filtration rate (GFR) in late pregnancy or in women with preeclampsia [45].

**Figure 1:**
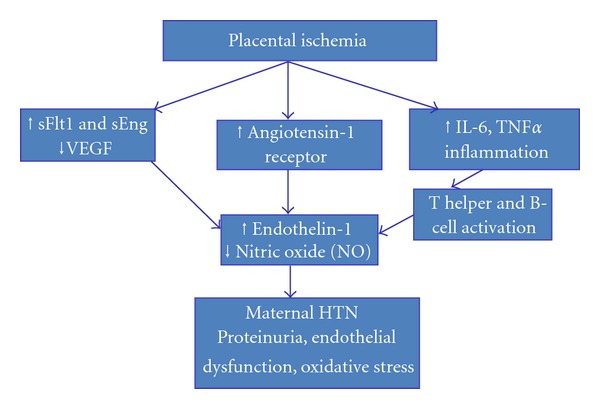
Showing unified hypothesis on pathogenesis of endothelial dysfunction, hypertension, and edema with preeclampsia.

Figure 1 illustrates how placental ischemia activates the preeclampsia cascade and alters the balance between endothelin-1 and NO, resulting in the observed endothelial dysfunction, hypertension, and proteinuria.

## 5. The Kidney in Preeclampsia: New Concepts on Pathogenesis

The phenotypic effects of maternal endothelial damage have been best understood in the kidney, where glomerular endotheliosis, generalized swelling, and vacuolization of endothelial cells have been noted [46, 47]. It appears that VEGF plays a critical role in maintenance of normal glomerular endothelial integrity. Furthermore, *in vitro* angiogenesis studies have demonstrated that exogenous VEGF/PlGF or an antibody against sFlt-1 can reverse the antiangiogenic effects of preeclamptic plasma [44]. It has been shown that podocyte injury and reduced specific podocyte protein expressions contribute to proteinuria in preeclampsia. In a recent study, Wang et al. showed that urinary excretion of podocyte specific proteins, such as, podocalyxin, nephrin, and Big-h3 were significantly elevated in women with preeclampsia [48].

## 6. Therapies for Preeclampsia: Magnesium**** Sulfate, Aspirin, Calcium, Antioxidants, ****Endothelin Antagonists

Currently the main therapy for preeclampsia is to deliver the baby as soon as he/she is most prudent to enhance maternal and fetal wellbeing.

### 6.1. Magnesium Sulfate

This is the most effective agent in prevention of eclampsia in women with preeclampsia. It has antiseizure effects as well as a being a vasodilator. Studies have shown that magnesium sulfate decreases pulsatility index in uterine, umbilical, and fetal arteries in women with preeclampsia [48, 49]. However, the mechanism of seizure prevention in preeclampsia may be independent of changes in angiogenic factors, as one study did not find alteration in angiogenic factor levels in preeclamptic women given magnesium sulfate, compared to placebo controls [50]. Magnesium sulfate also normalizes placental interlekin-6 secretion in a model of preeclampsia [51], which supports the fact that some of its benefits may drive from anti-inflammatory actions.

### 6.2. Aspirin

Since inflammation appears to play a significant role in the pathogenesis of preeclampsia, some investigators have studied the role of aspirin in prevention and therapy of preeclampsia. While some small single-center studies suggest a benefit for aspirin in preeclampsia [52, 53], larger multicenter trials have shown little or no effect [54, 55]. Possible explanations for the lack of reproducibility of benefits in larger studies include heterogeneity of preeclampsia, with benefit of therapy evident only in small subsets, as well as possible publication bias in favor of positive results in small studies. Dudley et al. performed a meta-analysis which has suggested a possible benefit of antiplatelet agents in preeclampsia. A large meta-analysis showed that aspirin use reduced the incidence of preeclampsia by 17%, and a 14% reduction in fetal and neonatal deaths, with 72 women needing treatment to benefit one woman [56, 57]. It is possible that benefits from aspirin in prevention of preeclampsia and its vascular complication may derive not just from an anti-inflammatory action but from a putative effect of restoring the balance between thromboxane and prostacyclin in the vasculature. Before using aspirin to prevent preeclampsia, consideration must be given to the toxicity in the gastrointestinal tract and effects on renal function [58].

### 6.3. Calcium

 Calcium supplementation has been suggested by some to have modest benefits in prevention of preeclampsia [59]. However, a large, randomized trial by Levine failed to find any benefit of supplemental calcium (2 gm daily) given to women in early pregnancy in prevention of preeclampsia [60]. Another randomized, controlled trial by the WHO showed that while calcium supplementation did not reduce the incidence of preeclampsia, it appeared to reduce adverse outcomes in women who developed preeclampsia [59]. Consequently, calcium supplementation has been recommended in populations with a low calcium intake [61].

### 6.4. Antioxidants

 In response to data suggesting that increased oxidative stress and derangement of antioxidative mechanisms occur in preeclampsia [62], some have advocated use of antioxidants in treatment of preeclampsia. As with aspirin and calcium, small studies suggested a benefit, but the largest study to date done by Roberts et al showed no benefit [63]. With regards to Vitamin A and E, one study showed low levels in diabetics with preeclampsia [64], while another study showed low levels of vitamin A and E in preeclamptic women in northern Nigeria [65]. A multicenter, randomized, double-blind trial of vitamin C and E supplementation in early pregnancy in high-risk women by the World Health Organization (WHO) did not prevent preeclampsia [66]. Consistent with these observations, a recent systematic review of antioxidant administration in early pregnancy to prevent preeclampsia found no benefit [67].

### 6.5. Endothelin Antagonists

Since endothelin nitric oxide appears to act through endothelin to aggravate sFLt1-induced preeclampsia-like phenotype, some investigators have studied the benefits of endothelin antagonism in treatment of preeclampsia. Antagonism of endothelial-A receptor has proved beneficial in some animal models of preeclampsia. In one model, administration of ambrisentan, an endothelin antagonist, was shown to improve creatinine clearance and podocyte effacement in ENOS-deficient mice sFlt1 mice [68]. Treatment with Ambrisentan also decreased proteinuria and ameliorates the endotheliosis classically seen in the kidneys of preeclamptic animals [69]. However, use of endothelin antagonists in early human pregnancy has not been tried as they have also been shown to induce birth defects in some studies [70]. It is possible that these agents may have a better safety profile if used in late pregnancy, but more studies would be needed to establish their safety in this setting. The goal remains to find therapeutic and preventive measures that can reduce maternal and fetal mortality from preeclampsia [71].

### 6.6. Use of Nitric Oxide Donors

Since preeclampsia is associated with reduced synthesis of vasodilators and increased synthesis of vasoconstrictors, researchers have sought to investigate possible therapeutic benefit for use of NO donors in treatment and prevention of preeclampsia. Animal data have shown that chronic nitric oxide blockade is associated with hypertension, proteinuria, and reductions in kidney function [72, 73]. Consistent with these observations, use of nitric oxide donors has shown benefit in a few human studies [74–77]. In one case a successful pregnancy followed use of nitric oxide donors in a patient with scleroderma who had had preeclampsia in a preceding pregnancy [77]. However, until this is validated in large scale, prospective randomized studies use of these nitric oxide donors cannot be recommended in all patients. They should be considered in selected patients, such as, those with scleroderma [78].

## 7. Unresolved Issues in Pathogenesis

### 7.1. Postpartum Preeclampsia/Eclampsia and the Antiangiogenic Theory

Postpartum preeclampsia (occurrence of hypertension and proteinuria) or postpartum eclampsia (occurrence of hypertension, proteinuria, and seizures) after delivery challenges the concept of the primacy of placental ischemia as the critical determinant of the occurrence of this syndrome. In this situation, the placenta has been removed, thus eliminating the source of the antiangiogenic factors that creates the milieu that helps trigger the vascular cascade. While postpartum eclampsia may occur from progression of preeclampsia, it may rarely occur without preceding preeclampsia and posing a diagnostic challenge [79]. Larsen has described factors that increase the risk for postpartum preeclampsia, such as, a body mass index (BMI) > 30, antenatal hypertensive disease, cesarean delivery, and African American race [78]. More education of providers and patients may help prevent late postpartum eclampsia [79].

### 7.2. Role of Thromboxane Synthase

Since 1985, it has been known that abnormal hemostasis and coagulopathy occur in preeclampsia and eclampsia [80]. This appeared to be related to an imbalance between an increased vasoconstrictor (thromboxane) and a decreased vasodilator (prostacyclin) in maternal blood [81]. Consistent with these observations, an increase in thromboxane synthase has been observed in decidua and trophoblast cells of placenta of women with preeclampsia. Recently some investigators have shown that reduced methylation of thromboxane synthase gene is correlated with its increase of vascular expression in preeclampsia [82]. How this altered thromboxane synthase gene expression plays into the deranged antiangiogenic milieu remains to be established. One putative link could be that reduced DNA methylation may increase thromboxane synthase in in neutrophils that infiltrate maternal blood vessels triggering inflammation in vascular smooth muscle and endothelium. This culminates in endothelial dysfunction, hypertension, and edema.

## 8. Conclusion

An accumulating body of evidence is helping to elucidate the pathogenesis of preeclampsia, showing the intricate link between placental and vascular ischemia, impaired angiogenesis, vascular inflammation, and endothelial dysfunction. If perturbed endotheil-1 signaling and/or disruption of endothelin-1/NO balance are proven to be the final common pathway for induction of preeclampsia, this concept would have huge translational potential implications. Translational studies are now needed to show how this emerging concepts can be applied to facilitate early diagnosis and therapy.
